# Evidence for the roles of oxidative stress, nitrosative stress and Nf-Kb activation in Tenofovir Disoproxil Fumarate (TDF) induced renal damage in rats

**DOI:** 10.1186/1471-2334-12-S1-P6

**Published:** 2012-05-04

**Authors:** Hemalatha Ramamoorthy, Bina Issac, Premila Abraham

**Affiliations:** 1 Department of Biochemistry, Christian Medical College, Bagayam, Vellore- 632 002, Tamilnadu, India; 2 Department of Anatomy, Christian Medical College, Bagayam, Vellore- 632 002, Tamilnadu, India

## Background

Nephrotoxicity is a dose limiting side effect of Tenofovir (TDF), a commonly used anti-HIV drug. About 37-45% of patients on anti-HIV drugs suffer from renal damage. As the mechanism of pathogenesis of TDF induced renal damage is not clear, it is necessary to elucidate the mechanism in order to prevent the renal damage. This study investigates the roles of nitroso-oxidative stress and NFkB activation in TDF induced renal damage.

## Methods

Rats were administered 2 daily doses of TDF (300mg/kg body weight) by gavage for 35 consecutive days, control rats received water alone. On the 36^th^ day, the rats were sacrificed, and the kidneys were used for histological examination, immunohistochemical analysis, and assay of the activities of antioxidant enzymes, myeloperoxidase and NFkB. Data were analyzed with Mann Whitney U test.

## Results

TDF administration to the rats resulted in renal damage. Electron microscopically, damage to the mitochondria of the proximal tubules was observed. Statistically significant increase in protein carbonyl content and nitrate levels (p<0.008), decrease in reduced glutathione (61%, p<0.01), protein thiol (33%, p<0.03), and activities of the antioxidant enzymes was observed. A 16 fold increase in the activity of NFkB (p <0.05), and a 9 fold increase in myeloperoxidase activity (p<0.01) were observed in the kidneys of TDF treated rats. The renal tissues of TDF treated rats stained strongly for nitrotyrosine and PARP.

## Conclusion

Nitroso-oxidative stress and NFkB activation contribute to TDF induced renal damage in rats. The source of these free radicals may be the damaged mitochondria and /activated neutrophils.

